# The Intersection Between *Schistosoma mansoni* Infection and Dyslipidemia Modulates Inflammation in the Visceral Adipose Tissue of Swiss Webster Mice

**DOI:** 10.3390/tropicalmed10080217

**Published:** 2025-07-31

**Authors:** Thainá de Melo, Isadora do Monte Silveira Bruno, Luciana Brandão-Bezerra, Silvia Amaral Gonçalves da Silva, Christiane Leal Corrêa, Luciana Silva Rodrigues, José Roberto Machado-Silva, Renata Heisler Neves

**Affiliations:** 1 Romero Lascasas Porto Laboratory of Helminthology, Department of Microbiology, Immunology and Parasitology, Medical Sciences College, Rio de Janeiro State University, Rio de Janeiro 20550-170, Brazil; thainamelo029@gmail.com (T.d.M.); isasilveira6@gmail.com (I.d.M.S.B.); lulithomas@gmail.com (L.B.-B.); jrmasilva@gmail.com (J.R.M.-S.); 2 Laboratory of Parasitic Immunopathology, Medical Sciences College, Rio de Janeiro State University, Rio de Janeiro 20550-170, Brazil; silvasag@gmail.com; 3 Department of Pathology and Laboratories, Medical Sciences College, Rio de Janeiro State University, Rio de Janeiro 20550-170, Brazil; christiane.leal@gmail.com; 4 Laboratory of Immunology, Department of Pathology and Laboratories, Medical Sciences College, Rio de Janeiro State University, Rio de Janeiro 20550-170, Brazil; lrodrigues.uerj@gmail.com

**Keywords:** *Schistosoma mansoni*, high-fat diet, dyslipidemia, adipocyte remodeling, inflammation

## Abstract

**Background:** Dyslipidemia and schistosomiasis are major public health challenges, particularly in endemic regions where their coexistence may influence host metabolism and immune responses. This study aimed to evaluate visceral adipose tissue (AT) remodeling in a murine model of acute *Schistosoma mansoni* infection combined with diet-induced dyslipidemia. **Methodology:** Female Swiss Webster mice were fed either a standard or high-fat diet (HFD) for 29 weeks and infected with *S. mansoni* at week 20. Nine weeks after infection, biochemical, morphometric, histopathological, and immunological analyses were performed. **Results:** The HFD promoted weight gain and dyslipidemia, while *S. mansoni* infection alone did not alter lipid profiles but partially mitigated the metabolic effects of the HFD. Morphometric analysis revealed adipocyte hypertrophy and reduced cell number in HFD-fed animals. In HFD-fed infected mice, infection partially reversed hypertrophy, suggesting a modulatory effect on AT remodeling. Histopathological examinations showed that while a HFD induced mild inflammation, infection led to intense leukocyte infiltration, hyperemia, and plasma cell degeneration. Peritoneal lavage confirmed a proinflammatory immune profile. **Conclusions:** These findings indicate that the interaction between a HFD and *S. mansoni* infection exacerbates adipose tissue inflammation and metabolic alterations, highlighting the complex interplay between parasitic infection, diet, and immune-metabolic regulation.

## 1. Introduction

Schistosomiasis is a parasitic disease caused by trematode worms of the genus *Schistosoma*, manifesting in acute or chronic forms [1]. This helminth infection affects over 251.4 million people worldwide, with endemic regions spanning 78 countries, placing an estimated 700 million individuals at risk [2]. In Brazil, *Schistosoma mansoni* is the predominant species, with transmission closely linked to socioeconomic conditions [3].

The pathogenesis of *S. mansoni* infection is shaped by the parasite–host interaction, and metabolic disorders may further disrupt this balance [4]. Notably, lipid-rich environments enhance parasite fertility and immune evasion, exacerbating disease severity in individuals with dyslipidemia [5].

Excessive fat intake from a high-fat diet (HFD) leads to lipid accumulation in the bloodstream and expansion of adipose tissue depots [6]. Adipose tissue plays a key role in energy storage, preventing lipid accumulation in non-adipose tissues [7]. However, its regulation extends beyond energy intake and expenditure. Lipolytic activity in adipose tissue is tightly linked to leptin secretion, a key hormone involved in the regulation of energy balance and metabolic homeostasis [8]. Leptin promotes mitochondrial lipid oxidation, ensuring efficient energy utilization [9]. Over time, excessive fat intake contributes to insulin resistance and chronic low-grade inflammation, as adipocyte hypertrophy triggers macrophage recruitment and inflammatory signaling [10,11].

The interplay between *S. mansoni* infection and HFD-induced dyslipidemia remains an emerging area of research. Given that schistosomiasis can induce metabolic alterations in the host, it is critical to investigate how this parasitic infection interacts with diet-induced adipose tissue remodeling. Understanding these effects is especially important in endemic regions, where individuals are often affected by both conditions, which may exacerbate metabolic imbalances and disease progression.

## 2. Materials and Methods

### 2.1. Experimental Design

Newly weaned female Swiss Webster mice (21 days old) were housed under standard laboratory conditions in the bioterium of the Department of Microbiology, Immunology, and Parasitology, Rio de Janeiro State University (FCM-UERJ). Environmental conditions were maintained at a temperature of 22 ± 2 °C, relative humidity of 60 ± 10%, and a 12:12-h light/dark cycle, with ad libitum access to food and water. All procedures were conducted in accordance with the ethical guidelines of the Ethics Committee on the Care and Use of Experimental Animals (CEUA-UERJ) and complied with Brazilian regulations on animal use in research and education [12,13]. The study was approved under protocol No. 028/2018 by CEUA-UERJ, Brazil. Every effort was made to minimize animal suffering throughout the experiment.

**Figure 1:**
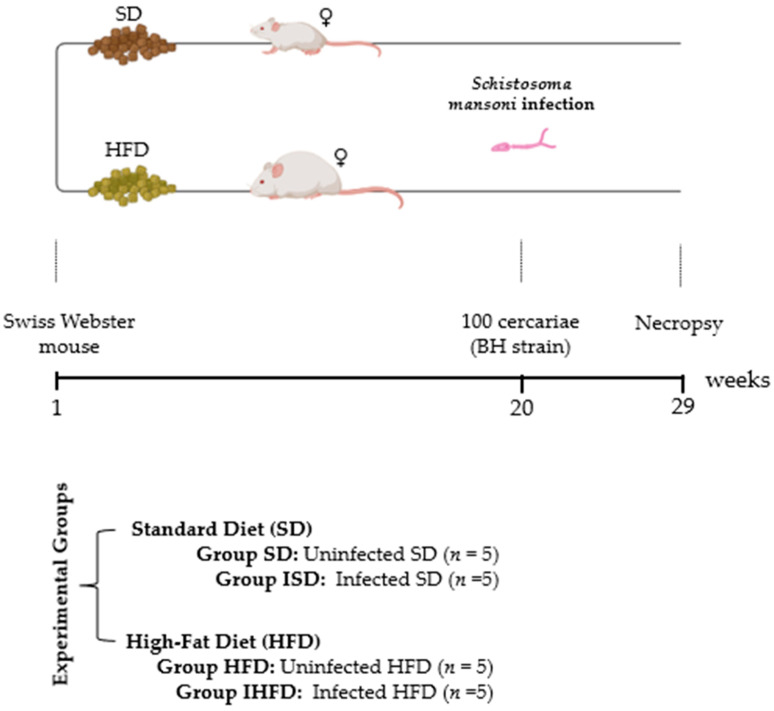
Experimental design. Female Swiss Webster mice were fed either a high-fat diet (HFD) or a standard diet (SD) for 29 weeks. At week 20, the animals were subcutaneously infected with 100 cercariae of *Schistosoma mansoni* (BH strain). After 9 weeks of infection, the animals were euthanized in a CO_2_ chamber and subsequently necropsied.

The mice were randomly assigned to groups receiving either a standard chow diet (Nuvilab CR1—NUVITAL Nutrients Ltda., Colombo, Paraná, Brazil), comprising 60% carbohydrates, 28% protein, and 12% lipids (4.6 kcal/g body wt./day) for laboratory animals, or a high-fat diet (PRAG Soluções, São Paulo, Brazil), containing 47% carbohydrates, 24% proteins, and 29% lipids (5.7 kcal/g body wt./day), as previously described [14]. After 20 weeks on their respective diets, half of the animals fed the standard or high-fat diet were subcutaneously infected with 100 *S. mansoni* cercariae (BH strain), obtained from *Biomphalaria glabrata* provided by the National Reference Laboratory—Malacology (LRNEM/IOC/FIOCRUZ, Rio de Janeiro, Brazil). Body mass progression was assessed by weighing the animals weekly using a digital analytical scale (Marte AL500C, São Paulo, Brazil) with a precision of 0.01 g. The experimental animals were randomly divided into four groups (each *n* = 5): SD—uninfected, standard diet; HFD—uninfected, high-fat diet; ISD—infected, standard diet; and IHFD—infected, high-fat diet. After 29 weeks (9 weeks of infection), the animals were euthanized in a carbon dioxide (CO_2_) chamber (Figure 1).

### 2.2. Biochemical Analyses

Blood samples were collected via cardiac puncture on the day of euthanasia, following a 10 h fasting period, without performing thoracotomy. Heparin was used as an anticoagulant, and plasma was separated by centrifugation at 3500× *g* for 15 min to obtain cell-free plasma, free of hemolysis. The lipid fractions measured included Total Cholesterol (TC), Triglycerides (TG), and High-Density Lipoprotein (HDL) using semi-automatic biochemical equipment (BIOCLIN 100, Quibasa Química Básica, Belo Horizonte, MG, Brazil) following the manufacturer’s instructions. Very Low-Density Lipoprotein (VLDL) and Low-Density Lipoprotein (LDL) values were determined using the Friedewald formula [15]. Non-HDL cholesterol was calculated by subtracting the HDL value from the total cholesterol [16].

### 2.3. Processing, Morphometric Analysis, and Histopathological Evaluation of Visceral Adipose Tissue

**Figure 2:**
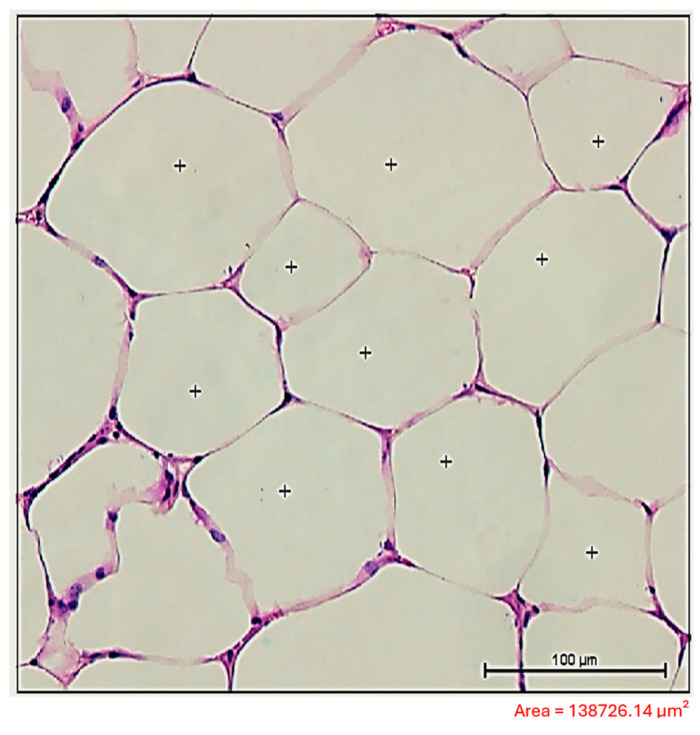
Bright-field light microscopy of histological sections of adipocytes from uninfected mice fed a high-lipid diet. (+) Adipocytes validated according to the criteria established by the author. Adipose tissue sections were stained with Hematoxylin and Eosin and visualized under a bright-field microscope using a 20× objective (total magnification: 200×). Scale bar: 100 μm.

The visceral adipose tissue was fixed in 10% buffered formalin for 24 h before undergoing routine histological processing and paraffin embedding [17]. Sections of 5 μm thickness were cut using a microtome (Leica RM2125 RTS, Leica Biosystems, Nussloch, Germany) for subsequent staining with Hematoxylin and Eosin (H&E). Histopathological examination was performed using a microscope (Olympus BX53) equipped with a camera (Olympus SN 4M04717, U-TV0.35XC-2, Tokyo, Japan), and images were captured using Olympus cellSens Entry image analysis software (version 1.18). Morphometric analysis of the adipose tissue was conducted using a Nis-Elements image analysis program (version 3.22.00) with a bright-field microscope (Nikon Eclipse 80i, Tokyo, Japan). At 20× objective (200× amplification), measurements of the area, perimeter, and diameter of 10 adipocytes in 10 random fields per animal were taken. These fields were selected randomly within the visceral adipose tissue, ensuring that adipocytes were completely contained within the area pre-established (Figure 2) by the authors (≅138,726.14 μm^2^) [17,18].

### 2.4. Peritoneal Macrophage Isolation and Stimulation

On the day of euthanasia, peritoneal cells were harvested by intraperitoneal lavage with 5 mL of cold (4 °C) RPMI 1640 medium (Cultilab, São Paulo, Brazil). Following gentle abdominal massage (30 s), the exudate was carefully aspirated using the same syringe and maintained on ice until processing. Under sterile conditions in a laminar flow hood, the collected fluid was transferred to pre-labeled 15 mL conical tubes. The cell concentration was adjusted to 2 × 10^6^ cells/mL in RPMI 1640 medium supplemented with 10% heat-inactivated fetal bovine serum (FBS) and 1% L-glutamine (Cultilab, São Paulo, Brazil), and the cells were incubated in a CO_2_ incubator at 37 °C in a humidified atmosphere containing 5% CO_2_. After incubation, the supernatants containing nonadherent cells were discarded, and the adherent macrophage monolayers were stimulated with lipopolysaccharide (LPS, 1 μg/mL) for 48 h. Supernatants were collected and stored at −80 °C until cytokine measurements.

### 2.5. Isolation and In Vitro Stimulation of Splenic Lymphocytes

Splenic lymphocytes were isolated by mechanical dissociation of spleen tissue on a 1 mm nylon mesh in 5 mL of RPMI 1640 medium. The cell suspension was transferred to 15 mL conical tubes and centrifuged at 458× *g* for 5 min at 4 °C. The pellet was treated with ACK lysis buffer to remove erythrocytes, followed by two washes with RPMI medium under the same centrifugation conditions. Cells were counted using a Neubauer hemocytometer and adjusted to 2 × 10^6^ cells/mL in RPMI medium supplemented with 10% FBS. A total of 500 µL of the cell suspension was plated per well in 24-well plates, and 1 µL of Concanavalin A (Con A) was added to stimulate proliferation. Plates were incubated at 35 °C with 5% CO_2_ for 48 h. Supernatants were then collected and stored at −80 °C for cytokine analysis.

### 2.6. Quantification of Cytokines

The concentrations of cytokines from peritoneal macrophages and splenic lymphocytes were quantified using the Cytometric Bead Array (CBA) method. Two kits from BD Biosciences (Franklin Lakes, NJ, USA) were used: the Mouse Th1/Th2/Th17 Cytokine Kit (Cat. No. 560485), which detects IL-2, IL-4, IL-6, IFN-γ, TNF, IL-17A, and IL-10; and the Mouse Inflammation Kit (Cat. No. 552364), which measures IL-6, IL-10, MCP-1, IFN-γ, TNF, and IL-12p70. Samples were incubated with a combination of fluorescent beads coated with specific antibodies against each cytokine (labeled with allophycocyanin, APC)- and phycoerythrin (PE)-tagged detection antibodies for 2 h at room temperature. Following incubation, the samples were washed with the manufacturer’s washing solution. Approximately 10.000 bead events per sample were recorded using a FACS Canto II flow cytometer (Becton Dickinson, Mountain View, CA, USA) running the FACS Diva software (version 3.0.1). Analysis was performed with FCAP Array software (BD Biosciences), and cytokine concentrations were calculated based on standard curves ranging from 20 to 5000 pg/mL, following the manufacturer’s guidelines.

### 2.7. Statistical Analysis

Statistical analyses were performed using GraphPad Prism software, version 5. First, a normality test was conducted to assess whether the data followed a Gaussian distribution. Subsequently, comparisons between groups were made using the one-way ANOVA test, followed by the Student–Newman–Keuls post hoc test. A significance level of *p* ≤ 0.05 was considered statistically significant. Data are presented as mean ± standard deviation for each experimental group from two independent experiments (*n* = 5). The experimental groups were compared as follows: the effect of diet (SD and HFD), the effect of infection in animals fed a standard diet (SD and ISD), the effect of infection in animals fed a high-fat diet (HFD and IHFD), and the combined effect of diet and infection (ISD and IHFD).

## 3. Results

### 3.1. Effect of High-Fat Diet and Schistosoma mansoni Infection on Body Mass Progression

**Figure 3:**
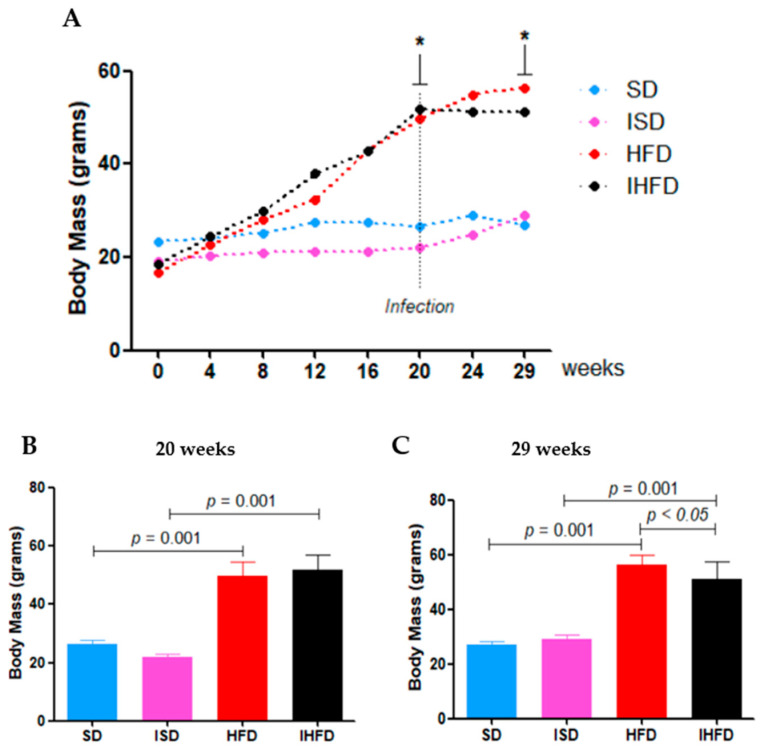
Body mass trajectory in mice subjected to high-fat diet and *Schistosoma mansoni* infection: (**A**) Body mass progression (expressed in grams) of Swiss mice throughout the 29-week experimental protocol; (**B**) Body mass milestone after 20 weeks; (**C**) Final body mass recorded at the end of the experiment. * Significant difference *p* < 0.05. One-way ANOVA test with Student–Newman–Keuls post hoc test. Abbreviations: uninfected, standard diet (SD); infected, standard diet (ISD); uninfected, high-fat diet (HFD); infected, high-fat diet (IHFD).

Body mass progression is shown in Figure 3. After 20 weeks, animals in the two HFD group (49 ± 5 g) had an 88% increase (*p* = 0.001) compared to the SD group (26 ± 1 g). In the infected group, IHFD animals (51 ± 5 g) showed a 135% increase (*p* = 0.001) relative to ISD animals (22 ± 1 g). No other comparisons showed significant differences (*p* > 0.05) at this time point. At the end of the experiment (week 29), animals in the HFD group (56 ± 3 g) maintained a 110% increase (*p* = 0.001) in body mass compared to the SD group (27 ± 1 g). The IHFD group (51 ± 6 g) showed a 76% increase in body mass compared to the ISD group (29 ± 1 g; *p* = 0.001) and a 9% reduction compared to the HFD group (56 ± 3 g; *p* = 0.01). The high-fat diet promoted a sustained increase in body mass throughout the experiment in both infected and non-infected animals. However, *Schistosoma mansoni* infection partially mitigated this effect, as evidenced by the lower body mass observed in the IHFD group compared to the HFD group.

### 3.2. Impact of High-Fat Diet and Schistosoma mansoni Infection on Lipid Profile: Modulatory Effects and Dyslipidemic Changes

The biochemical results are presented in Table 1. Total cholesterol levels were higher in the HFD group (+80%; *p* = 0.001) compared to the SD group, confirming the dyslipidemic effect of the high-fat diet. No significant difference was found between the ISD and SD groups (*p* > 0.05), indicating that infection alone did not alter this parameter. In contrast, the IHFD group (+56%, *p* = 0.01) showed elevated cholesterol levels compared to the ISD group. Interestingly, a reduction in total cholesterol was observed in IHFD (−25%, *p* = 0.01) animals compared to the HFD group, suggesting a partial modulatory effect of *Schistosoma mansoni* infection under conditions of dietary lipid excess.

The HFD group presented higher triglyceride levels (+59%; *p* < 0.05) compared to the SD group, indicating a pronounced effect of the high-fat diet on triglyceride accumulation. Infection alone and SD did not result in significant alterations (*p* > 0.05). An increase in triglyceride levels was observed in the IHFD group (+34%, *p* < 0.05) compared to the ISD group. However, no significant difference was found between IHFD and HFD groups, reinforcing the predominant role of the diet in triglyceride accumulation.

Animals in the HFD group showed higher levels of HDL (+84%, *p* < 0.05), VLDL (+59%, *p* < 0.05), LDL (+84%, *p* = 0.01), and non-HDL cholesterol (+80%, *p* = 0.001) compared to the SD group. The IHFD group showed higher levels of HDL (+44%, *p* > 0.05), VLDL (+34%, *p* < 0.05), LDL (+63%, *p* > 0.05), and non-HDL cholesterol (+57%, *p* < 0.05) compared to the ISD group. However, when comparing the IHFD and HFD groups, reductions were observed in HDL (−36%, *p* < 0.05), LDL (−26%, *p* < 0.05), and non-HDL cholesterol (−23%, *p* < 0.05). No significant differences were observed between the ISD and SD groups (*p* > 0.05).

The results indicate that the high-fat diet led to an increase in lipid levels, including HDL, VLDL, LDL, and non-HDL cholesterol, compared to the SD, confirming the dyslipidemic effect of the HFD. Infection with *S. mansoni* (IHFD) further elevated certain lipid parameters, particularly VLDL, LDL, and non-HDL cholesterol, compared to the ISD. However, when comparing IHFD and HFD groups, the infection seemed to modulate lipid levels, as reductions were observed in HDL, LDL, and non-HDL cholesterol, suggesting that the presence of *S. mansoni* partially mitigated the effects of the high-fat diet.

### 3.3. Cellular Mechanisms of Adipose Tissue Remodeling: Interplay Between High-Fat Diet and Schistosoma mansoni Infection

**Figure 4:**
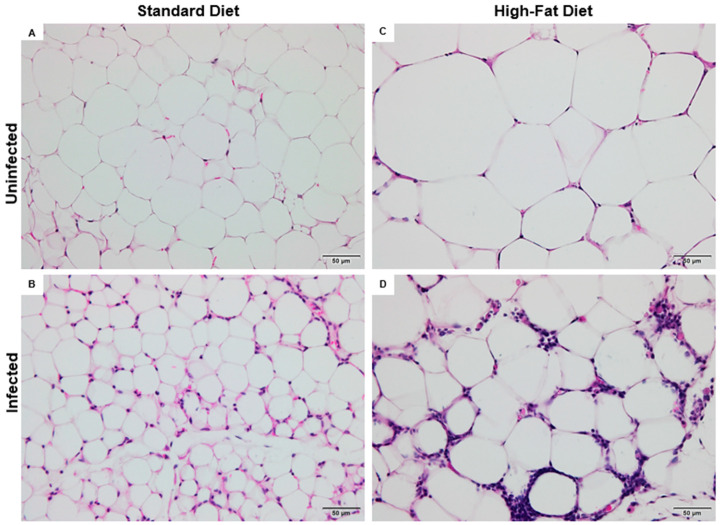
Cellular mechanisms of adipose tissue remodeling: interplay between high-fat diet and *Schistosoma mansoni* infection. (**A**) Histological section of visceral adipose tissue from uninfected mice fed a standard diet; (**B**) Histological section of visceral adipose tissue from *Schistosoma mansoni*-infected mice fed a standard diet; (**C**) Histological section of visceral adipose tissue from uninfected mice fed a high-fat diet; (**D**) Histological section of visceral adipose tissue from *Schistosoma mansoni*-infected mice fed a high-fat diet. Adipose tissue sections were stained with hematoxylin and eosin. Scale bar: 50 μm.

The morphometric data of adipose tissue is presented in Table 1 and Figure 4. The ISD group exhibited a 174% increase in the number of adipocytes per field compared to the SD group (*p* < 0.001). In contrast, the HFD group showed a 59% reduction relative to SD (*p* < 0.001), reflecting the influence of the high-fat diet on adipose tissue cellularity. Among the infected animals, IHFD presented an 82% decrease when compared to ISD (*p* < 0.001). No significant difference was found between IHFD and HFD (*p* > 0.05), suggesting that *S. mansoni* infection did not further impact the adipocyte number when combined with a high-fat diet.

Compared to the SD group, animals in the ISD group showed reductions in cell size, with decreases of 30% in diameter, 27% in perimeter, and 51% in area (*p* < 0.001 for all). In contrast, the HFD group exhibited increased adipocyte dimensions relative to SD, with gains of 11% in diameter, 17% in perimeter, and 31% in area (*p* < 0.001 for all). When comparing the infected groups, IHFD animals displayed marked hypertrophy compared to ISD, with increases of 47% in diameter, 52% in perimeter, and 132% in area (*p* < 0.001). However, IHFD group animals showed reductions of 7% in diameter (*p* < 0.001), 7% in perimeter (*p* < 0.01), and 5% in area (*p* < 0.001) in comparison to the HFD. These results suggest that *S. mansoni* infection alone leads to smaller adipocytes, while a high-fat diet promotes hypertrophy. Interestingly, in the context of dietary lipid excess, infection appears to partially counteract adipocyte enlargement, indicating a potential modulatory effect on adipose tissue remodeling (Table 2).

### 3.4. Histopathological Alterations in the Adipose Tissue of Mice Subjected to a High-Fat Diet and Schistosoma mansoni Infection

**Figure 5:**
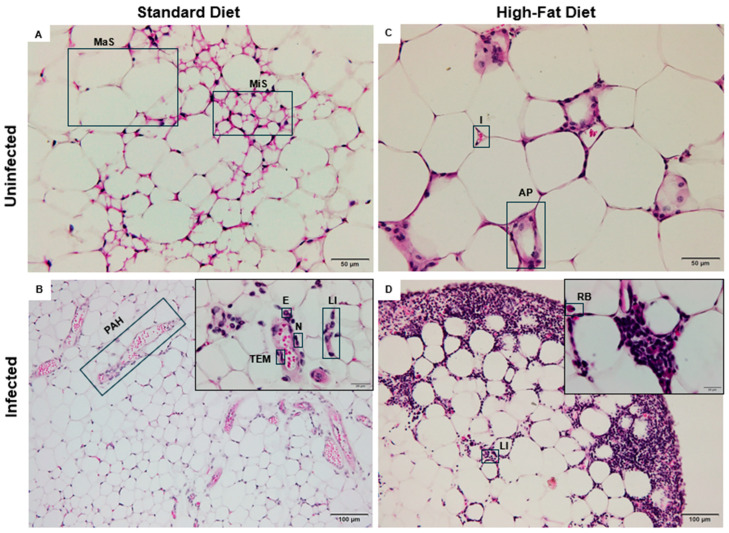
Histopathological alterations in the adipose tissue of mice subjected to a high-fat diet and *Schistosoma mansoni* infection. (**A**) Histological section of visceral adipose tissue from uninfected mice fed a standard diet; (**B**) Histological section of visceral adipose tissue from *Schistosoma mansoni*-infected mice fed a standard diet; (**C**) Histological section of visceral adipose tissue from uninfected mice fed a high-fat diet; (**D**) Histological section of visceral adipose tissue from *Schistosoma mansoni*-infected mice fed a high-fat diet. Abbreviations: (AP) apoptotic adipocytes; (E) eosinophils; (I) ischemia; (LI) leukocyte infiltration; (Mas) macrovesicular steatosis; (MiS) microvesicular steatosis; (N) neutrophils; (PAH) pathological active hyperemia; (RB) Russell bodies; (TEM) transendothelial migration. Adipose tissue sections were stained with hematoxylin and eosin. Scale bars: (**A**,**C**) = 50 μm; (**B**,**D**) = 100 μm and 20 μm.

The normal visceral adipose tissue is predominantly composed of spherical adipocytes, whose thin cytoplasmic borders enclose a single lipid droplet in the form of triglycerides. These droplets may appear as microvesicles or macrovesicles, reflecting the cell’s lipid storage capacity. This characteristic histological pattern was observed in mice from the control group fed a SD (Figure 5A).

The ISD group showed marked alterations in the cellular architecture of visceral adipose tissue, indicating a significant inflammatory process. Diffuse leukocyte infiltration was observed, composed of polymorphonuclear cells such as neutrophils, eosinophils, and macrophages, distributed throughout different areas of the tissue. In addition, pathological active hyperemia was identified, characterized by vascular dilation and increased local blood flow, consistent with the inflammatory response triggered by *S. mansoni* infection (Figure 5B).

Adipocyte hypertrophy was evident in the HFD group, resulting in capillary remodeling likely due to ischemia. Impaired blood flow may have compromised tissue perfusion, leading to apoptosis of some adipocytes. Consequently, scattered macrophage clusters were found throughout the adipose tissue, indicating a low-grade inflammatory response (Figure 5C).

In the IHFD group, adipocyte hypertrophy was observed—a feature already identified in HFD. However, the cellular architecture exhibited a highly inflamed profile, with abundant leukocyte infiltrates dispersed throughout the visceral adipose tissue. Histopathological evaluation revealed the presence of plasma cells with hyaline degeneration, known as Russell bodies, characterized by rounded cells with a homogeneous pink appearance (Figure 5D).

### 3.5. Impact of Schistosoma mansoni Infection and Metabolic Status on Macrophage Cytokine Production

**Figure 6:**
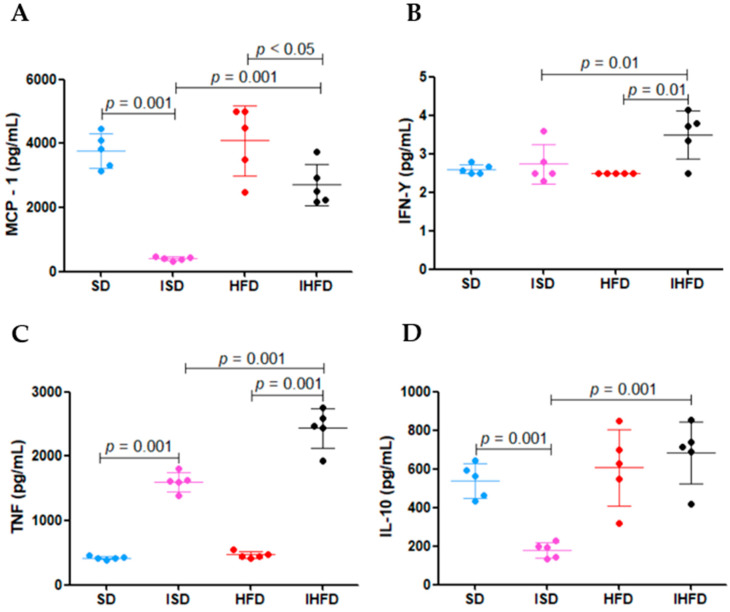
Impact of *Schistosoma mansoni* infection and metabolic status on macrophage cytokine production. Panels: (**A**) Monocyte Chemoattractant Protein-1 (MCP-1); (**B**) Interferon-gamma (IFN-γ); (**C**) Tumor Necrosis Factor (TNF); (**D**) Interleukin-10 (IL-10). Abbreviations: uninfected, standard diet (SD); infected, standard diet (ISD); uninfected, high-fat diet (HFD); infected, high-fat diet (IHFD). Cytokine concentrations were measured using the Mouse Inflammation CBA Kit (BDTM), and the detection limits for each cytokine were as follows: MCP-1 (52.7 pg/mL), IFN-γ (2.5 pg/mL), TNF (7.3 pg/mL) and IL-10 (17.5 pg/mL). One-way ANOVA test with Student–Newman–Keuls post hoc test.

The cytokine levels expressed by peritoneal macrophages are presented in Figure 6.

Schistosomal infection led to a decrease (−89%; *p* = 0.001) in MCP-1 cytokine expression in the ISD group (399 ± 61 pg/mL) compared to the SD group (3774 ± 61 pg/mL) (Figure 6A). In contrast, the IHFD group (2711 ± 648 pg/mL) showed a marked increase (+579%; *p* = 0.001) in MCP-1 levels relative to the ISD group. However, MCP-1 expression in the IHFD group was 34% lower (*p* < 0.05) than in the HFD group (4093 ± 1092 pg/mL). These findings indicate that schistosomal infection suppresses MCP-1 expression in animals fed a standard diet, while a high-fat diet partially reverses this suppression, suggesting an interaction between the inflammatory profile and the host’s metabolic state.

Animals in the IHFD group (3.5 ± 0.6 pg/mL) showed an increase in IFN-γ levels, with rises of 40% and 28% (*p* = 0.01) compared to the HFD (2.5 ± 0 pg/mL) and ISD (2 ± 0.5 pg/mL) groups, respectively, as shown in Figure 6B. These results suggest that a high-fat diet promotes increased systemic inflammation by stimulating macrophages and lymphocytes to produce pro-inflammatory cytokines.

Schistosomal infection in the ISD group (1605 ± 149 pg/mL) increased TNF levels by 280% (*p* = 0.001) compared to the SD group (421 ± 29 pg/mL). The IHFD group (2433 ± 311 pg/mL) showed increases of 419% and 52% (*p* = 0.001), respectively, compared to the HFD (468 ± 51 pg/mL) and IS groups (Figure 6C). The results suggest that *S. mansoni* infection increases TNF levels, and that a high-fat diet further amplifies this inflammatory response.

The ISD group (180 ± 39 pg/mL) showed a 67% reduction in IL-10 levels (*p* = 0.001) compared to the SD group (538 ± 89 pg/mL). In contrast, the IHFD group (682 ± 160 pg/mL) exhibited a 279% increase (*p* = 0.001) compared to the ISD group, as shown in Figure 6D. Acute *S. mansoni* infection in this study indicates that the Th2 response is still adjusting, as evidenced by the reduced IL-10 levels in the ISD group. In contrast, the high-fat diet restored IL-10, possibly as a compensatory mechanism against the chronic low-grade inflammation induced by the diet. The acute *S. mansoni* infection in this study indicates that the Th2 response is still adjusting, as evidenced by the reduced IL-10 levels in the ISD group. In contrast, the high-fat diet restored IL-10, possibly as a compensatory mechanism against the chronic low-grade inflammation induced by the diet.

### 3.6. Modulation of Splenocyte Cytokine Production by Diet and S. mansoni Infection

**Figure 7:**
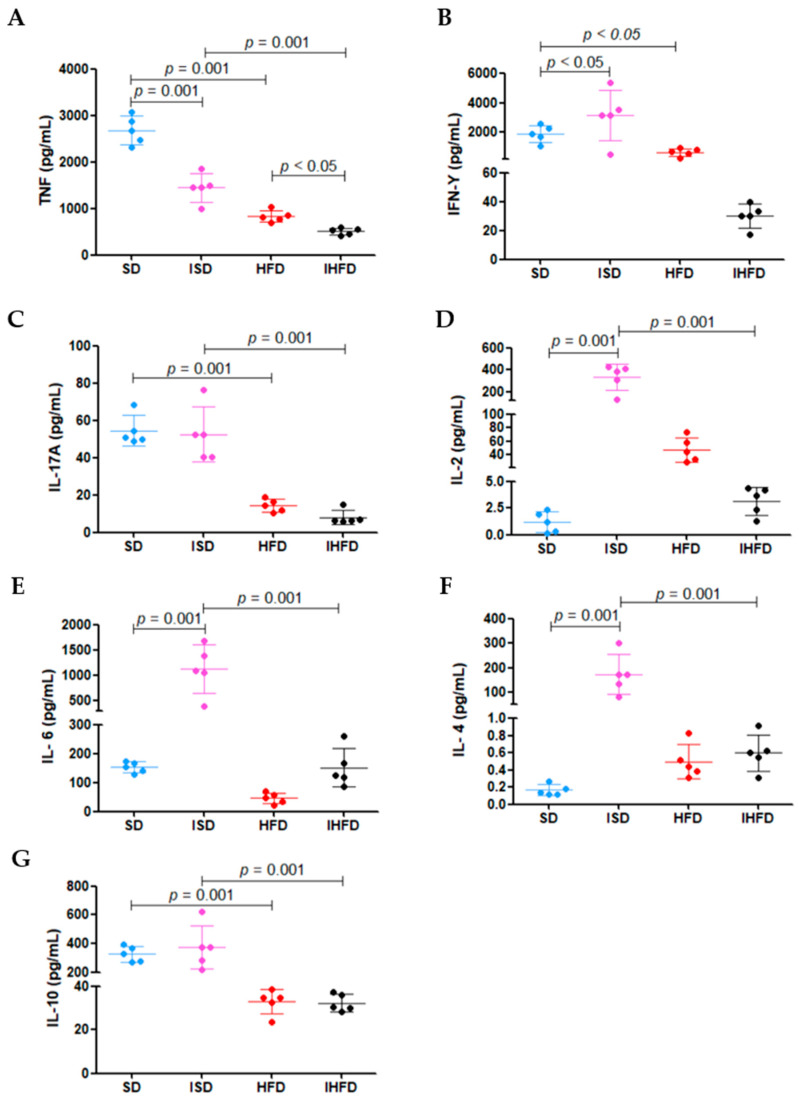
Modulation of splenocyte cytokine production by diet and *S. mansoni* infection. Panels: (**A**) Tumor Necrosis Factor (TNF); (**B**) Interferon-gamma (IFN-γ); (**C**) Interleukin-17A (IL-17A); (**D**) Interleukin-2 (IL-2); (**E**) Interleukin-6 (IL-6); (**F**) Interleukin-4 (IL-4); (**G**) Interleukin-10 (IL-10). Abbreviations: uninfected, standard diet (SD); infected, standard diet (ISD); uninfected, high-fat diet (HFD); infected, high-fat diet (IHFD). Cytokine concentrations were measured in splenocyte culture supernatants after mitogenic stimulation using the BDTM CBA Mouse Th1/Th2/Th17 Cytokine Kit. The detection limits for each cytokine were as follows: TNF (0.9 pg/mL), IFN-γ (0.5 pg/mL), IL-17a (0.8 pg/mL), IL-2: (0.1 pg/mL), IL-6 (1.4 pg/mL), IL-4 (0.03 pg/mL) and IL-10 (16.5 pg/mL). One-way ANOVA test with Student–Newman–Keuls post hoc test.

The results of cytokine production by splenocytes following mitogenic stimulation with Con A are presented in Figure 7.

The high-fat diet led to a 69% reduction in TNF levels in the HFD group (834 ± 127 pg/mL) compared to the SD group (2880 ± 304 pg/mL; *p* = 0.001). Schistosomal infection also decreased TNF production, with a 46% reduction in the ISD group (1447 ± 307 pg/mL) compared to the SD group (*p* = 0.001). Notably, the IHFD group (508 ± 72 pg/mL) exhibited an even more pronounced decrease, with TNF levels reduced by 65% compared to the ISD group (*p* = 0.001) and 39% in the group HFD (*p* < 0.05), as shown in Figure 7A.

The IFN-γ (Figure 7B) production was increased by 64% in the ISD group (3146 ± 1712 pg/mL) compared to the SD group (1915 ± 581 pg/mL; *p* < 0.05). In contrast, animals in the HFD group (677 ± 253 pg/mL) showed a 65% reduction in IFN-γ levels relative to the SD group (*p* < 0.05). The increased IFN-γ in the ISD group indicates macrophage activation during the acute phase of *S. mansoni* infection. The high-fat diet in the HFD group reduced this response, potentially impairing parasite control.

The high-fat diet caused a 74% decrease (*p* = 0.001) in IL-17a levels in the HFD group (14 ± 3 pg/mL) compared to the standard diet group (SD: 54 ± 8 pg/mL). A similar reduction pattern (−85%; *p* = 0.001) was observed in the IHFD group (8 ± 3 pg/mL) relative to the ISD group (52 ± 14 pg/mL), as shown in Figure 7C. These results suggest that a high-fat diet impacts the inflammatory response in the animals.

In the ISD group, there was a marked increase in IL-2 (+28%; 333 ± 121 pg/mL; Figure 7D)**,** IL-6 (+635%; 1127 ± 477 pg/mL; Figure 7E) and IL-4 (+194%; 172 ± 72 pg/mL; Figure 7F) levels compared to the SD group (1 ± 0.9 pg/mL; 153 ± 19 pg/mL; 0.1 ± 0.05 pg/mL, respectively; *p* = 0.001). In contrast, the IHFD group showed a sharp reduction in these cytokines compared to the ISD group: IL-2 (−99%; 3 ± 1 pg/mL), IL-6 (−87%; 152 ± 67 pg/mL) and IL-4 (−100%; 0.6 ± 0.1 pg/mL). The IL-10 levels (Figure 7G) increased modestly in the ISD group (+15%; 376 ± 151 pg/mL) compared to the SD group (327 ± 54 pg/mL) but dropped by 91% in the IHFD group (32 ± 4 pg/mL) compared to the ISD group (*p* = 0.001). These results suggest that acute *S. mansoni* infection stimulates splenocyte cytokine production, whereas a high-fat diet suppresses this response, even in the presence of infection.

## 4. Discussion

A high-fat diet induces a progressive and sustained increase in body mass and is associated with metabolic dysfunctions, including insulin resistance and chronic low-grade inflammation [19]. In contrast, *Schistosoma mansoni* infection has been shown to attenuate weight gain, likely due to immune system activation and increased energy expenditure driven by the chronic inflammatory response [20]. This modulation appears to involve alterations in the signaling pathways of pro- and anti-inflammatory cytokines, such as IL-10 and TNF-α, which influence lipid metabolism and energy homeostasis [4,21]. Furthermore, the parasite may impair intestinal nutrient absorption and alter the gut microbiota, thereby contributing to changes in body composition [22]. These findings highlight the complexity of the interaction between nutritional and parasitic factors in metabolic regulation. Chronic infections like schistosomiasis may exert a partial protective role against both the primary and secondary effects of excessive dietary fat intake [20,22,23].

In our study, the lipid profile analyses confirmed the detrimental metabolic impact of a high-fat diet, reflected by elevated atherogenic lipoproteins. However, the presence of *S. mansoni* significantly attenuated this lipid accumulation, particularly in animals exposed to excessive dietary fat [24]. This modulation may result from the infection-driven inflammatory response, which increases host energy expenditure and alters lipid handling dynamics [25]. These findings suggest that, even in a metabolically adverse environment, schistosomiasis exerts a partial protective effect against diet-induced dyslipidemia.

Through histopathological and morphometric analyses, we characterized visceral adipose tissue remodeling. Our data revealed that *S. mansoni* infection induces an inflammatory response in visceral adipose tissue, regardless of diet. In the context of high-fat intake, we observed adipocyte hypertrophy along with reduced hyperplasia, likely reflecting impaired adipogenesis. In animals subjected to both a high-fat diet and infection, inflammation was accompanied by increases in adipocyte diameter, perimeter, and area—indicative of exacerbated hypertrophy. A possible explanation lies in the reduced efficiency of adipogenesis under lipid overload, where a substantial portion of circulating lipids are diverted to hepatic oxidation, reducing substrate availability for adipose tissue storage [26,27].

Interestingly, in our results, infected animals fed a standard diet (ISD) exhibited marked adipocyte hyperplasia. This finding may be associated with elevated plasma leptin levels, a hormone secreted by adipose tissue that plays a crucial role in regulating appetite, energy balance, and host defense [28]. Parasitic infections can increase or decrease leptin levels depending on the type of parasite and the host’s immune response [29]. In this context, elevated leptin during infection may contribute to adipocyte remodeling, promoting both cellular hypertrophy and hyperplasia [30]. Furthermore, plasma leptin levels correlate directly with total adipose tissue mass and are closely associated with the recruitment of new adipocytes [8]. This relationship establishes a feedback loop in which hyperleptinemia stimulates adipogenesis through the upregulation of leptin mRNA expression in adipocytes [31].

Schistosomiasis also induces significant metabolic alterations in adipose tissue. The inflammatory response triggered by the infection enhances TNF-mediated lipolysis, contributing to reductions in both adipocyte number and volume [4,21]. Notably, *S. mansoni* does not synthesize lipids but relies on host-derived cholesterol and triglycerides for its survival and reproduction [32]. This lipid acquisition is crucial for parasite development, as it supports egg production and granuloma formation in the host liver [33].

Adipose tissue functions as an active endocrine organ by secreting adipokines that mediate communication with immune cells and regulate energy balance and lipid metabolism [34]. Excessive lipid intake enhances adipokine release and promotes the recruitment of immune cells, particularly macrophages, into adipose depots [35]. This immune cell infiltration amplifies local inflammation through the secretion of pro-inflammatory mediators such as TNF, IL-6, and MCP-1 [11]. In our study, the reduced expression of the pro-inflammatory cytokine MCP-1 observed in infected animals can be attributed to the immunomodulatory effects induced by *S. mansoni* infection. During the establishment of the Th2 immune response, there is an increase in cytokines such as IL-4, IL-5, and IL-10, which act by suppressing inflammatory pathways through the inhibition of pro-inflammatory cytokines [36]. Among these, IL-10 plays a key regulatory role by downregulating the expression of chemokines like MCP-1, thereby limiting the recruitment of monocytes/macrophages to affected tissues [37]. Additionally, the infection promotes macrophage polarization toward the M2 phenotype, which is associated with anti-inflammatory functions and reduced production of mediators such as MCP-1 [38]. This more tolerogenic immune profile helps to mitigate tissue damage caused by the inflammatory response to parasite eggs, representing an important host-protective mechanism against excessive inflammation. However, it is important to highlight that although *S. mansoni* infection predominantly induces a Th2 response with anti-inflammatory effects [36], the concomitant presence of a diet rich in saturated fats intensifies immune activation, promoting a pro-inflammatory profile [39]. In this context, a high-fat diet stimulates the production of inflammatory cytokines, including IFN-γ, by macrophages and CD4+ Th1-type T lymphocytes [39].

Finally, studies report that *S. mansoni* infection can promote metabolic balance in hyperlipidemic hosts by reducing adipose tissue mass and attenuating adipocyte hypertrophy [40]. Taken together, these findings suggest that the metabolic changes induced by *S. mansoni*, particularly under high-fat dietary conditions, may reflect an adaptive host–parasite interaction capable of modulating adipose tissue remodeling. These insights offer a deeper understanding of the immunometabolic consequences of chronic helminth infections and suggest potential translational relevance for conditions such as obesity and dyslipidemia.

## 5. Conclusions

This study shows that a high-fat diet induces dyslipidemia and adipose tissue remodeling characterized by hypertrophy and inflammation. *Schistosoma mansoni* infection modulates these effects by reducing lipid accumulation and altering adipocyte characteristics. However, the interaction between a high-fat diet and *S. mansoni* infection exacerbates adipose tissue inflammation and metabolic alterations, highlighting the complex interplay between parasitic infection, diet, and immune-metabolic regulation. These findings emphasize the multifaceted nature of immunometabolic mechanisms that influence metabolic disorders, suggesting potential protective yet intricate roles of parasitic infections in diet-induced metabolic dysfunction.

## Figures and Tables

**Table 1:** Impact of high-fat diet and *Schistosoma mansoni* infection on the lipid profile.

Biochemical (mg/dL)	Study Groups			
	Standard Diet		Hyperlipidic Diet	
	SD	ISD	HFD	IHFD
Total cholesterol	107 ± 21	94 ± 14	194 ± 28 *	146 ± 22 ^$&^
Triglycerides	79 ± 9	87 ± 10	125 ± 35 *	117 ± 22 ^$^
Non-HDL cholesterol	94 ± 18	83 ± 14	169 ± 36 *	130 ± 23 ^$&^
LDL	78 ± 18	65 ± 15	144 ± 39 *	107 ± 24 ^&^
HDL	13 ± 4	11 ± 2	25 ± 9 *	16 ± 3 ^&^
VLDL	16 ± 2	17 ± 2	25 ± 7 *	23 ± 4 ^$^

Significant difference *p* ≤ 0.05 compared to groups: SD (*); ISD (^$^); HFD (^&^). One-way ANOVA test with Student–Newman–Keuls post hoc test. Abbreviations: uninfected, standard diet (SD); infected, standard diet (ISD); uninfected, high-fat diet (HFD); infected, high-fat diet (IHFD); High-Density Lipoprotein (HDL); Very Low-Density Lipoprotein (VLDL); Low-Density Lipoprotein (LDL); Non-High-Density Lipoprotein cholesterol (non-HDL). Values are expressed as mean ± SD.

**Table 2:** Cellular mechanisms of adipose tissue remodeling: interplay between high-fat diet and *Schistosoma mansoni* infection.

Parameters	Study Groups			
	Standard Diet		Hyperlipidic Diet	
	SD	ISD	HFD	IHFD
Adipocyte count per field	39 ± 10	107 ± 23 *	16 ± 4 *	19 ± 4 ^#^
Diameter	61 ± 16	43 ± 7 *	68 ± 24 *	63 ± 23 ^#$^
Perimeter (µm)	205 ± 57	149 ± 27 *	239 ± 85 *	226 ± 83 ^#$^
Area (µm^2^)	3127 ± 1595	1542 ± 591 *	4099 ± 2883 *	3585 ± 2559 ^#$^

Significant difference *p* ≤ 0.05 compared to groups: SD (*); ISD (^#^); HFD (^$^). One-way ANOVA test with Student–Newman–Keuls post hoc test. Abbreviations: uninfected, standard diet (SD); infected, standard diet (ISD); uninfected, high-fat diet (HFD); infected, high-fat diet (IHFD). Values are expressed as mean ± SD.

## Data Availability

The data generated or analyzed during this study are included in the article. Further details will be made available upon request.

## Abbreviations

The following abbreviations are used in this manuscript:

AT	Adipose Tissue
HFD	High-Fat Diet
SD	Standard Diet
S. mansoni	Schistosoma mansoni
